# Virological and Serological Discordant Profiles in COVID-19 Pneumonia: Two Atypical Clinical Cases

**DOI:** 10.3389/fimmu.2020.580867

**Published:** 2020-10-02

**Authors:** Martina Ranzenigo, Claudia Pastori, Gabriel Siracusano, Elena Pariani, Caterina Uberti-Foppa, Lucia Lopalco

**Affiliations:** ^1^Clinic of Infectious Diseases, San Raffaele Scientific Institute, Milan, Italy; ^2^Division of Immunology, Transplantation and Infectious Diseases, San Raffaele Scientific Institute, Milan, Italy; ^3^Department of Biomedical Sciences for Health, University of Milan, Milan, Italy; ^4^Vita-Salute San Raffaele University, Milan, Italy

**Keywords:** COVID-19, SARS-CoV-2, viral RNA, PCR, antibodies, neutralizing antibodies, NP, spike protein

## Abstract

Severe acute respiratory syndrome coronavirus 2 (SARS-CoV-2) infection is primarily diagnosed through viral RNA positivity in nasopharyngeal swabs, and it is associated with the early detection of specific immunoglobulins to SARS-CoV-2 proteins. We describe two moderate coronavirus disease 2019 (COVID-19) patients with WHO score 4/5 at the time of hospitalization, pneumonia, and oxygen saturation <94% and with a strong discrepancy between viral RNA and antibodies to SARS-CoV-2. One patient was positive for viral RNA but completely negative for binding and neutralizing antibodies, whereas the second patient was negative for viral RNA but with high levels of both neutralizing and binding antibodies. This observation is relevant to better understand the pathogenesis of this novel infection.

## Introduction

Severe acute respiratory syndrome coronavirus 2 (SARS-CoV-2) is a novel airborne coronavirus causing a high-consequence infectious disease [coronavirus disease 2019 (COVID-19)]. The World Health Organization (WHO) has declared on March 11, 2020, the novel coronavirus outbreak as a pandemic. One of the relevant questions regarding the current COVID-19 pandemic is the heterogeneity of the illness (1). The detection of viral RNA in upper and lower respiratory tract specimens is the basis of COVID-19 laboratory confirmation; for a COVID-19 case to be considered negative, two consecutively negative results of viral RNA detection taken 24 h apart are required (2). The S protein of SARS-CoV-2, which is classified as a class I viral fusion protein, is a protruded trimer on the viral envelope. Each spike monomer consists of an N-terminal S1 ectodomain and a membrane-proximal S2 ectodomain, which mediate receptor binding and membrane fusion, respectively (3). Another relevant protein is the nucleocapsid protein, a structural and immunogenic protein (4). Viral infection stimulates the immune system to produce specific antibodies. SARS-CoV-2-specific immunoglobulin M (IgM) are generated 3–5 days after onset of symptoms, and remain at a high level, thus suggesting the persistence of the acute phase of infection. IgG titer increases in the recovery stage rather than in the acute stage and can be elicited concomitantly with IgM (5, 6). Moreover, IgA and IgG production against SARS-CoV-2 develops mainly in severe COVID-19, thus suggesting different grades of antibody responses associated with progression of the diseases (7).

## Clinical Case Presentation

In this report, two COVID-19 cases with atypical virological and serological SARS-CoV-2 profiles are discussed. The clinical characteristics of the two patients are shown in Table 1. In detail, Patient #1 is a 66-year-old female, without previous comorbidities. She was diagnosed with SARS-CoV-2 infection and hospitalized with a mild pneumonia (left posterobasal patchy ground-glass opacity), without other symptoms (except occasional cough). After admission, the patient was given oxygen through a nasal cannula. *Legionella* and pneumococcal urinary antigen tests and serology for acute infection of *Mycoplasma pneumoniae* and *Chlamydophila pneumoniae* were negative. Laboratory workup performed at hospital admission and at subsequent times revealed a normal white blood cell count and absolute lymphocyte count; C-reactive protein (CRP), interleukin (IL)-6, and D-dimer remained in range during the hospitalization. RT-PCR was positive for SARS-CoV-2 RNA in three nasopharyngeal swabs collected in a range of 21 days. She was discharged in stable condition after 3 weeks of hospitalization; two consecutive nasopharyngeal swabs were negative to SARS-CoV-2 RNA 4 weeks after symptom onset.

Patient #2 is a 77-year-old man who presented to the emergency department with dyspnea and cough. His comorbidities included arterial hypertension, *trans-*catheter ablation of atrial fibrillation, and right hemisphere ischemic stroke. Chest CT demonstrated patchy ground-glass opacity in the left middle lung field subsequently followed by extensive lesions in both lungs that implied severe hypoxemia for which he started continuous positive airway pressure (CPAP) therapy. As opposed to Patient #1, laboratory workup revealed many COVID-19 pneumonia markers as high white blood cell count of 17.5 × 10^9^/L with reduced absolute lymphocyte count of 0.4 × 10^9^/L, high D-dimer of 10.80 μg/ml (normal value ≤0.8 μg/ml), high CRP 79.6 mg/L (normal value ≤6 mg/L), high IL-6 of 4,814 pg/ml (normal value <7 pg/ml), and high ferritin of 2,904 ng/ml (normal value <400 ng/ml). During hospitalization, his inflammatory markers down-trended, which correlated with symptomatic improvement, and he was discharged in stable condition after a total of 7 weeks of hospitalization. At hospital admission, the patients had a high WHO score 4 and 5 for Patient #1 and Patient #2, respectively (8), while at discharge, the score was 1 for both of them. Both patients gave informed consent.

## Detection of SARS-CoV-2 RNA by RT-PCR

The identification of SARS-CoV-2 in nasopharyngeal swabs collected from Patient #1 was carried out by homemade one-step real-time RT-PCR following the WHO guidelines. After total RNA extraction through a commercial kit (QIAGEN, Germany), a one-step real-time RT-PCR targeting different portions of SARS-CoV-2 N gene was performed (9). Three consecutive nasopharyngeal swabs collected in a range of 21 days (from day 1 to day 21 from onset of symptoms) were positive to SARS-CoV-2 RNA detection.

In Patient #2, RT-PCR was negative for SARS-CoV-2 in five consecutive nasopharyngeal swabs collected in a range of 53 days (from day 4 to day 57 since symptom onset). The first two nasopharyngeal swabs of Patient #2 were analyzed by a homemade real-time RT-PCR as described by Corman et al. (10) targeting both E and RdNP genes. All swabs collected from both patients were analyzed for all time points by a commercial assay (Cobas^®^ 6800, Roche Molecular Diagnostics, Pleasanton, CA, United States) targeting both E and RdNP genes, according to manufacturer’s instructions. Commercial assay confirmed the results obtained with the homemade RT-PCR.

## SARS-CoV-2-Specific Serological Evaluations

Plasma samples from Patient #1 and Patient #2 were collected on day 32 from symptom onset. The samples were analyzed by using three different methods to evaluate IgM, IgG, and IgA specific to SARS-CoV-2 proteins. In detail, IgG, IgA, and IgM to nucleocapsid protein (NP) were measured through NOVATEC ELISA KIT according to the manufacturer’s instruction. IgG to Spike (S) protein (full-length ectodomain) was evaluated through LIAISON^®^ DiaSorin CLIA KIT. S1 ectodomain (S1)- and S2 ectodomain (S2)-specific IgG, IgA, and IgM were evaluated by homemade ELISA. Cutoff values were established to be 10 arbitrary units (AU) for NOVATEC ELISA kit, 12 AU for LIAISON^®^ DiaSorin Kit, and 10 AU for homemade ELISA. To establish the cutoff value of homemade ELISA, plasma samples from 40 healthy controls collected before the COVID-19 pandemic were used. Briefly, in the homemade ELISA, 0.1 μg/well of S1 or S2 protein (ABEOMICS) was coated on 96-well plates and incubated overnight at 4°C in 50 mM carbonate/bicarbonate buffer pH 9.5. The plates were blocked for 1 h at 37°C with 200 μl/well of PBS containing 10% BSA and 0.05% Tween 20. Then, each plasma was tested at 1:100 dilution in duplicate. After washing, 100 μl/well of horseradish peroxidase (HRP)-conjugated goat -anti human IgA, -anti human IgG, or -anti human IgM (Southern Biotech), diluted 1/6,000 in blocking buffer, was added to the plates and incubated for 30 min at 37°C. Fifty microliters/well of TMB 2C (KPL-SeraCare) was plated. The optical density (OD) values were read at wavelengths of 450 and 620 nm.

The results indicated the complete absence of the SARS-CoV-2-specific IgM, IgG, and IgA in Patient #1, as they resulted below cutoff value for all three classes of antibodies and with all the three different systems as shown in Table 2, although Patient #1 was positive for SARS-CoV-2 RNA detection by RT-PCR. Moreover, Patient #1 was antibody negative for two consecutive times, on day 32 as shown in Table 2 and on day 64 since symptom onset (data not shown), thus confirming the complete absence of SARS-CoV-2-specific antibodies. To verify that Patient #1 was not affected by hypogammaglobulinemia or agammaglobulinemia, the immunoglobulin quantification was evaluated during hospitalization and resulted within the normal range: 10.43 g/L.

Patient #2 showed high titers of both IgG and IgA with all three assays (homemade ELISA for S1 and S2, commercial ELISA for NP, commercial CLIA for S) and high levels of IgM to S1 and NP but not to S2, but negative for RT-PCR, as shown in Table 2. All results refer to plasma collected at 32 days from symptom onset. The antibody reactivity to both S1 and NP was higher than to S2, thus confirming that S2 is less immunogenic than S1 or NP (4). The titers of antibodies expressed as AUs were comparable to those observed in 28 COVID-19 patients matched for age, sex, and symptoms but positive for SARS-CoV-2 RNA detection (Tables 1, 2). Of note is that the median value for IgA to S2 was below cutoff, and the range was 5.5 to 22 AU, thus confirming the low immunogenicity.

**TABLE 1:** Characteristic of studied patients #1 and #2.

			WHO score		Clinical parameters				Laboratory parameters at hospital admission					
ID	Sex	Age	Admission	Discharged	Oxygen saturation on room air	HIGH Resolution CT (HRCT)	Hospitalization weeks	CPAP	White cell count	Absolute lymphocyte count	C-reactive protein	Ferritin	IL-6	D-dimer
									**n 4.8–10.8 × 10^9^/L**	**n 1–4.8 × 10^9^/L**	***n* < 6 mg/L**	***n* < 400 ng/mL**	***n* < 7 pg/mL**	***n* < 0.77 μg/mL**

Pat.#1	F	66	4	1	92%	Pneumonia	3	No	7.9	2.7	0.9	Not present	Not present	0.27
Pat.#2	M	77	5	1	55%	Pneumonia	7	Yes	17.5	0.4	79.6	2904	4814	10.8
Patients N28	16M/12F	median 71	range (4–5)	1	range (50–93%)	Pneumonia	range (3–8)	1F and 4M						
														

**TABLE 2:** SARS-CoV-2 specific RNA and binding and neutralizing antibodies in plasma from all studied patients.

		Antibody reactivity (AU)*			
ID	RT-PCR	IgG*	IgA*	IgM*	Neutralization
		S1 – S2 – NP – S	S1 – S2 – NP	S1 – S2 – NP	IC50**
Pat.#1	Positive	4.6 – 4.8 – 6.3 – <12	6.4 – 3.7 – 2	3.4 – 4.5 – 4.3	<60
Pat.#2	Negative	31 – 16 – 34 –>15	40.5 – 21 – 29.5	20 – 7.3 – 17	8100
Controls/16 Male	Positive	23.2 – 14 – 20.5 – ND***	28.6 – 12.8 – 18.2	17.5 – 5.6 – 18.2	850 (range <60–6800)
Controls/12 Female	Positive	20.6 – 15.2 – 22 – ND***	26.3 – 11 – 20.8	16.3 – 4.8 – 16.9	1160 (range <60–10622)

*AU stands for Arbitrary Unit. It has been calculated following the formula: median OD sample x10/cut off value. **IC50 stands for dilution of sera inhibiting 50% of infection. ***ND stands for not determined.

To evaluate the possible interference of antibodies to other respiratory pathogens, plasma from both patients was tested in ELISA for binding to *Mycoplasma*, *Chlamydia*, cytomegalovirus (CMV), adenovirus, parainfluenza, influenza A, influenza B, and respiratory syncytial virus (RSV). Both patients were negative for IgM to all tested antigens, and Patient #1 was positive for IgG to *Mycoplasma*, CMV, adenovirus, parainfluenza, influenza A, influenza B, and RSV but negative for *Chlamydia*. Patient #2 was IgG positive for *Mycoplasma*, *Chlamydia*, CMV, adenovirus, parainfluenza, influenza A, and RSV but negative for influenza B.

Moreover, pseudotyped SARS-CoV-2 virus micro neutralization assay was used to assess the neutralizing activity in plasma collected from both Patient #1 and Patient #2 at day 32 from symptom onset. An Env-defective HIV-1 backbone and an HIV-based lentiviral packaging system, both expressing the luciferase reporter gene, were used to produce pseudotyped particles bearing the full-length spike (S) protein of SARS-CoV-2 on the surface. In detail, HEK 293T/17 cells were co-transfected with Env-defective HIV backbone expressing firefly luciferase (pNL43R^–^E^–^-luciferase) and pcDNA3.1 expression vector encoding the S protein using FuGENE^®^ HD according to the manufacturer’s instruction. As a control, SARS-CoV-2 RSV was produced in the absence of the spike-encoding plasmid (Δ env). Viral titers were determined by transducing 10^4^ HEK 293T/17-ACE2/TMPRSS2 cells with two-fold serial dilutions of RSVs to each well of a 96-well titration plate. After 48 h incubation at 37°C 5% CO_2_, firefly luciferase expression was quantified by the Bright-Glo^TM^ assay luciferase system (Promega) and the VICTOR X Light Luminescence Plate Reader (PerkinElmer). Each relative luminescence unit (RLU) value obtained at different RSV dilution points was converted into RLU/ml, and the arithmetic mean of these concentrations was considered as the RSV production titer (expressed as RLU/ml). Plasma samples from 10 subjects collected prior to the emergence of SARS-CoV-2 were used as negative controls. Neutralization assays were performed by incubating 10^6^ RLU of pseudotyped viruses with endpoint two-fold serial dilutions of plasma samples at 37°C 5% CO_2_ for 1 h before addition of 10^4^ HEK 293T/17-ACE2/TMPRSS2 cells per well. After 48–72 h at 37°C, the cells were lysed and luciferase activity was measured as previously reported. Neutralization titers were expressed as IC50 values. As expected, Patient #1 did not show any neutralization, whereas Patient #2 shows a high level of infectivity reduction with an IC50 8.100 plasma dilution (Table 2), thus suggesting a possible correlation between binding and neutralizing antibodies. The plasma from 28 COVID-19 patients was evaluated in a neutralization assay at days ranging from 17 to 64 of onset, with a median of 35 from symptom onset, and the results were reported in Table 2.

## Discussion

The relationship between viral RNA positivity and the dynamics of serum antibody responses from COVID-19 patients is not fully elucidated yet.

Patient #1 had milder clinical symptoms than Patient #2, a SARS-CoV-2-positive carrying time of at least 21 days and absence of virus-specific IgM, IgG, and IgA antibodies 32 days after the onset of symptoms. It is unlikely that the absence of antibodies can be attributed to low sensitivity of the serological tests as three different assays were performed with three different SARS-CoV-2 proteins and all of them were negative. Of note, two close contacts of this patient with virologically documented asymptomatic infections had the same discordant viro–serological profile (SARS-CoV-2 RNA positive in nasopharyngeal swabs and negative antibodies 1 month after the first viral positivity), suggesting a possible viral role in the discordant profiles. We could hypothesize that virus, in these patients, has a higher level of glycosylation pattern, thus the immune system is not stimulated adequately to elicit SARS-CoV-2-specific antibodies. Unfortunately we cannot verify this hypothesis as we do not have residual samples to perform genetic analysis and/or phenotype prediction. Another crucial point is the role played by T cell immunity, which could be stronger in the two close contacts of Patient #1, as the two contacts were asymptomatic (data not shown). Moreover, the immunoglobulin level of Patient #1 was within the normal range, thus a deficit in the immunoglobulin repertoire is excluded. It is unclear whether the absence of SARS-CoV-2-specific antibodies in Patient #1 can result in a major susceptibility to reinfection, as reported in sporadic cases (11, 12). It has been reported that, in some cases, IgA response could be detected earlier than IgM response, although a high level of IgG is found (13). Another work shows that IgA has higher sensitivity than IgG (14). Nevertheless, these findings do not explain the complete absence of all classes of SARS-CoV-2-specific immunoglobulins in Patient #1.

We could hypothesize that in Patients #2, a rapid and deep viral infiltration in the tissues could result in the absence or low (under the limit of detection) viral load in the superior respiratory tract, but the brief and/or intermittent presence of virus could have been enough to allow antibody production. Alternatively, although unlikely, Patient #2 could be interpreted as a virological false negative, as reported by other investigators (15), suggesting that insufficient viral specimens and/or laboratory error might be responsible, although resulted negative to viral RNA detection in nasopharyngeal swabs for five consecutive times and with two different systems. Of note, in Patient #2, we found a primary immune response (IgM) for at least the more immunogenic proteins such as S1 and NP, so long time after disease onset. That could be interpreted as a long-lasting acute infection. Moreover, in agreement with literature data, patients with high titers of antibodies are associated with neutralizing activity (16). Another relevant observation is the subsequently greater severity of pneumonia in Patient #2 that can open questions whether the elicitation of antibodies is more strictly correlated to the severity of diseases and not related to viral RNA, although the antibody production has been shown in all cases of mild disease as well. Noteworthy, as for Middle East respiratory syndrome (MERS), patients with severe disease appear to have higher antibody titers than those with milder disease (17), although this latter finding does not explain the complete absence of antibody response, as all the hospitalized mild patients develop specific antibodies. Recently, asymptomatic SARS-CoV-2-positive patients have been shown to have lower antibody levels compared to symptomatic patients in the acute phase, with reduced IgG and neutralizing antibodies during the initial recovery phase. These data suggest that asymptomatic patients had a weaker immune response to SARS-CoV-2 infection, but once again, this finding does not explain the complete absence of antibodies in patients with mild disease (18). Indeed, it has been stated that 100% of COVID-19 patients develop IgG and IgM after a few days of symptom onset (6). Another point is the role played by immunoglobulins directed to other respiratory pathogens, which could interfere with SARS-CoV-2 immunoglobulins, but both patients did not have IgM to other pathogens and were IgG positive to several respiratory pathogens, thus it does not seem that these immunoglobulins may play any role in these two COVID-19 patients.

In our small group of patients (total 30 subjects), we found a strong discrepancy between viral RNA and elicitation of SARS-CoV-2-specific antibodies in only two patients (6.6%), but it could be interesting to evaluate larger numbers of COVID-19 patients to understand the biological mechanisms in SARS-CoV-2 infection and to better evaluate under epidemiological point of view whether these discrepancies may affect the analyses.

## Data Availability Statement

The raw data supporting the conclusions of this article will be made available by the authors, without undue reservation.

## Ethics Statement

The studies involving human participants were reviewed and approved by the Comitato Etico OSR, Milano. The patients/participants provided their written informed consent to participate in this study. Written informed consent was obtained from the individual(s) for the publication of any potentially identifiable images or data included in this article.

## Author Contributions

LL conceived and wrote the manuscript. MR and CU-F verified the clinical data. MR, CU-F, and EP wrote and revised the manuscript. CP, GS, and EP performed and analyzed all the tests. All authors contributed to the article and approved the submitted version.

## Conflict of Interest

The authors declare that the research was conducted in the absence of any commercial or financial relationships that could be construed as a potential conflict of interest.
